# Effects of Total Flavonoids of Epimedium on Bone Marrow Adipose Tissue in Ovariectomized Rats

**DOI:** 10.3389/fendo.2022.900816

**Published:** 2022-06-06

**Authors:** Lei Chen, Rui Ma, Peng Luo, Dan Shi, Xiao Shi, Hua Nian, Shi-Xin Chang, Wei Yuan, Guan-Wu Li

**Affiliations:** ^1^ Department of Radiology, Yueyang Hospital of Integrated Traditional Chinese and Western Medicine, Shanghai University of Traditional Chinese Medicine, Shanghai, China; ^2^ Department of Geriatrics, Yueyang Hospital of Integrated Traditional Chinese and Western Medicine, Shanghai University of Traditional Chinese Medicine, Shanghai, China; ^3^ Department of Pharmacy, Yueyang Hospital of Integrated Traditional Chinese and Western Medicine, Shanghai University of Traditional Chinese Medicine, Shanghai, China; ^4^ Department of Orthopaedics, Shanghai Fourth People’s Hospital Affiliated to Tongji University School of Medicine, Shanghai, China

**Keywords:** osteoporosis, total flavonoids of Epimedium, marrow adipocytes, white adipose tissue, brown adipose tissue, ovariectomy

## Abstract

Bone marrow adipose tissue has brown fat characteristics. Several studies have demonstrated that total flavonoids of Epimedium (TFE) could prevent bone loss and reduce the white adiposity in bone marrow induced by ovariectomy (OVX) in rats. However, the effects of TFE on marrow brown fat in OVX rats remain unclear. In this word, we addressed this question expected to provide a new target for preventing and treating osteoporosis. Thirty-six 3-month-old female Sprague-Dawley rats were equally divided into Sham controls, OVX controls, and OVX treated with TFE. Chemical shift coding magnetic resonance was performed to detect marrow fat fraction at the left femur at baseline, 6 and 12 weeks post-OVX. Bone mineral density at the lumbar spine and femur was measured by dual-energy x-ray absorptiometry. Serum bone biomarkers by ELISA, trabecular bone microarchitecture at the proximal tibia by micro-CT, quantitative parameters of marrow adipocyte by hematoxylin, and eosin staining were evaluated. The marrow adipocyte gene and protein expressions profile were determined by real-time quantitative PCR and immunostaining in whole tibiae. We found that TFE treatment could decrease bone turnover rate and improved bone mineral density and trabecular microarchitecture in OVX rats. OVX resulted in marrow adipogenesis as evidenced by increased marrow fat fraction, larger marrow adipocyte size, increased adipocyte number and percentage of adipocyte area, marrow white adipocyte gene, and protein expression, including PPARγ2 and FABP4. These pathological changes induced by estrogen deficiency were restored by TFE treatment. TFE also increased brown adipocyte expressions of the transcription factor Ucp1 and Prdm16 in whole tibiae. There was no detectible protein expression of brown adipocyte markers in the proximal tibia. Taken together, TFE regulation of bone marrow adiposity in OVX rats is mediated, at least in part, *via* maintaining the reciprocity of white and brown adipose tissue.

## Introduction

Bone marrow mesenchymal stem cells (MSCs) can differentiate into osteoblasts and adipocytes, which are delicately balanced for osteogenesis and adipogenesis during bone remodeling (1, 2). Growing evidence indicates that an increase in marrow adipogenesis inhibits osteoblastogenesis and promotes osteoclastogenesis. Therefore, a potential therapeutic target for treating estrogen deficiency-induced osteoporosis is to reduce the expansion of marrow adiposity (3, 4). Furthermore, a better understanding of the components of marrow fat tissue and its relationship with neighboring cells can contribute to a reasonable strategy when targeting marrow fat to prevent osteoporosis.

Adipocytes’ characteristics and anatomical structure are now widely recognized into three types: white, brown, and beige adipocytes (5). Excessive white adipose tissue in bone marrow is one of the pathogenic mechanisms of osteoporosis (6), while brown fat tissue, as an independent predictor of bone mass, could facilitate osteogenesis *via* regulating bone anabolism (7, 8). Interestingly, animal experiments showed that the brown-like phenotype of adipose tissue was found in the bone marrow cavity of mature mice, where bone remodeling is active (9–12). Moreover, the imbalance of brown and white fat in bone marrow may affect the balance of bone metabolism. It has been demonstrated that peroxisome proliferator-activated receptor-γ coactivator-1α could induce the formation of brown fat in the bone marrow MSCs and promote the browning of white fat (13) indicating the transformation between white and brown phenotypes. Therefore, we suspected that modulating the balance of bone marrow lipid metabolism to increase the brown fat content may provide new insight into preventing and treating osteoporosis.

The total flavonoids of Epimedium (TFE) are active components of Epimedium against osteoporosis (14). Previous studies indicated that TFE could promote osteogenic differentiation and inhibit adipogenic differentiation of MSCs (15–17). Previous *in vivo* studies also confirmed that the early treatment of TFE could prevent bone loss and reduce the white adiposity induced by estrogen deficiency (18). However, the exact mechanism of TFE on marrow adipose tissue, particularly brown fat, remains unclear. Since an association between osteogenesis and adipogenesis has been confirmed, and a mixed brown/white adipose tissue phenotype has been found in marrow adipose tissue, we hypothesized that TFE could maintain the reciprocity of white and brown adipose tissue in a rat model of estrogen deficiency-induced osteoporosis. To verify this hypothesis, in this study, we mainly observed the effects of TFE on bone marrow fat, especially brown fat markers, bone metabolic biomarkers, and bone trabecular microstructures in ovariectomized (OVX) rats, in expected to provide a new target for the prevention and treatment of osteoporosis.

## Materials and Methods

### Experimental Design

Thirty-six female Sprague-Dawley rats (3-month-old; average initial weight, 230 ± 15g) were used and housed in individual cages in an animal room. Room temperature was 22 ± 2°C with a 40-50% relative humidity and a 12 h light and 12 h dark cycle. Food and water were provided ad libitum. The rats were allowed to acclimatize for two weeks before being used for the study and were weighed every week throughout the experimental period. The rats were randomly divided into three groups with 12 per group :(1) sham-operation group (Sham), (2) ovariectomized group (OVX), and (3) OVX treated with TFE group (TFE, 191 mg/kg body weight/day) (Kanion Sunshine Pharmaceutical Co., Ltd., Jiangsu, China). The dosage of TFE was based on previous studies indicating that 191 mg/kg/day was sufficient to restore the bone mass and biomechanical strength to levels of the Sham group (19). All animal procedures followed the National Institutes of Health Guidelines on the Care and Use of Laboratory Animals. To reduce the number of rats used in the current study, we followed the recommendation of the Animal Ethics Committee to use only a standard daily dose of TFE. The Sham and OVX control rats were given the same volume of normal saline as the vehicle. The intervention started 3 days after surgery and lasted for 12 weeks. Administered the substances by daily intragastric gavage 6 times a week and adjusted the dose for the body weight of the rats once a week.

At the end of the 12th week, blood samples were collected by cardiac puncture under anesthesia and put 2mL serum into a sterile EP tube. After standing at 4°C for 24h, the serum was transferred into the sterile EP tube by low-temperature centrifugation (4°C, 3000rpm’5min) and stored at -80°C for biochemical analysis. At the end of treatment, the rats were killed by over injection of pentobarbital sodium. The L4 vertebral body, femur, and tibia were removed and protected by liquid nitrogen for later analysis.

### Enzyme-Linked Immunosorbent Assay (ELISA)

Serum 17β-estradiol levels (Kamiya Biomedical Company, Seattle, USA) and biomarkers of bone turnover were analyzed using enzyme-linked immunosorbent assays. Bone turnover biomarkers included bone formation marker type 1 collagen amino propeptide (P1NP) and bone resorption marker C-terminal telopeptides of type I collagen (CTX-I) (Immunodiagnostic Systems Nordic A/S, Denmark). All analyses were performed according to the manufacturer’s protocol.

### Dual-Energy X-Ray Absorptiometry (DXA)

The rat’s bone mineral density (BMD, g/cm^2^) was measured by a dual-energy X-ray absorptiometry (Hologic Inc., Bedford, MA) equipped with specific software for the small animal scanning mode. As previously described (20), the scanning regions were marked with respect to the femur and lumbar spine. Measurements were performed at baseline (before the OVX surgery) and after the interventions (6 and 12 weeks, respectively).

### Magnetic Resonance Imaging

**Figure 1:**
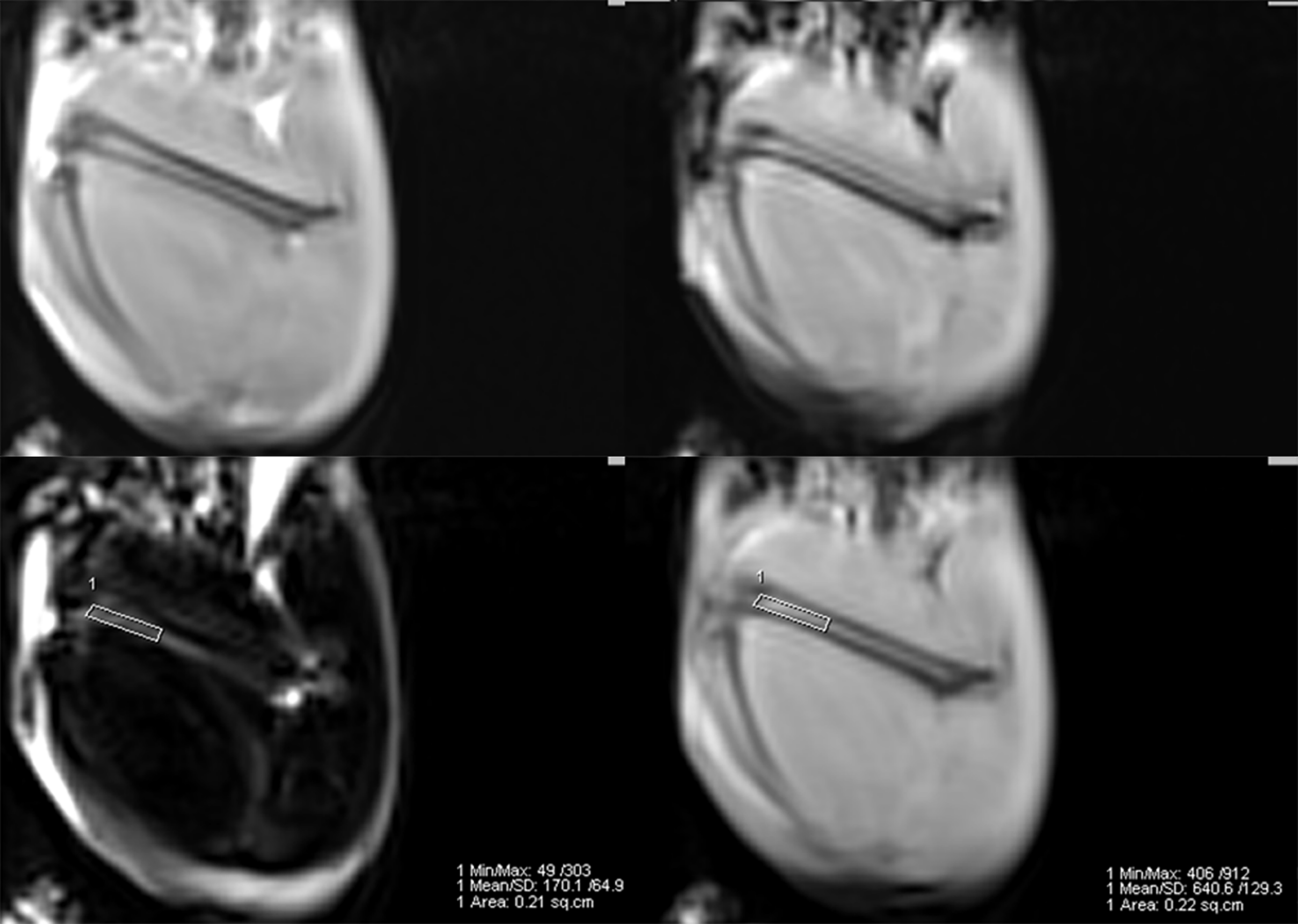
Example of a 2-point Dixon sequence for measuring fat fraction. The region of interest was manually drawn in the distal femur in a fat-only image, avoiding the cortical bone, and directly copied onto a water-only image.

MRI scans were conducted using a 3.0T MR scanner (Magnetom Verio, Siemens Healthcare, Germany), equipped with a 4-channel phased-array small animal coil (Shanghai Chenguang, CG-MUC18-H300-AS). In this protocol, A routine T1-weighted VIBE sequence (repetition time 5.91 ms; echo time 2.45/3.675 ms; flip angle 9°; matrix 192 × 192; slice thickness 1.5 mm) covering the entire femur was selected to measure bone marrow fat fraction (**Figure 1**). Fat fraction (FF) was calculated using the following formula as previously described (21, 22): FF (in %) = mean intensity*_fat image_*/(mean intensity*_fat image_* + mean intensity*_water image_*) ×100%. Imaging scans were performed at baseline (week 0), 6 and 12 weeks postoperatively to obtain the dynamic changes in marrow lipid content.

### Micro-Computed Tomography (Micro-CT)

The left tibia in each rat was measured with a micro-CT scanner (Xradia microCT-200A, ZEISS, Pleasanton, CA, USA) at 12.5-µm voxel resolution to assess trabecular microarchitectures. Microstructures of bone trabeculae, including bone volume fraction (BV/TV), trabecular separation (Tb. Sp), trabecular number (Tb. N) and trabecular thickness (Tb. Th), and structure model index (SMI), were determined according to guidelines, as described in detail elsewhere (22).

### Hematoxylin and Eosin Staining

After micro-CT scanning, left tibias were fixed in 10% buffered formalin for 24 h and then decalcified in 10% buffered ethylene diamine tetraacetic acid for 4 weeks. Specimens were embedded in paraffin, sectioned in 5 µm thick slices, and stained with hematoxylin and eosin. The photomicrographs were captured at a 200× magnification with five microscopic fields per section using a Leica DM6000B microscopy (Wetzlar, Germany). Image analysis, including adipocyte mean size (µm), adipocyte density (1/mm^2^), and percentage of adipocyte area (%) in all analyzed fields, was done using Image-Pro Plus software v6 (Media Cybernetics, Inc, Rockville, MD) as previously described (23). Samples were evaluated in a blinded fashion to avoid investigator bias.

### Immunohistochemistry and Immunofluorescence Staining

The bone immunohistochemistry was performed as described in detail elsewhere (24, 25). In brief, fatty acid-binding protein 4 (FABP4) and peroxisome proliferator-activated receptor gamma-2 (PPARγ2) immunostaining in paraffin-embedded 5-μm sections above mentioned were probed using an anti-FABP4 antibody (rabbit monoclonal to FABP4, Abcam) and anti-PPARγ2 antibody (Mouse monoclonal to PPAR gamma 2 + PPAR gamma, Abcam) immunohistochemistry/immunofluorescence Kits. For immunohistochemistry analysis of FABP4, heat-mediated antigen retrieval was performed using Tris/EDTA buffer. ImmunoHistoProbe one-step HRP Polymer was used as the secondary antibody. Hematoxylin was used as a counterstain. As a negative control, nonimmune mouse immunoglobulin G was used as the primary antibody. Pictures were obtained at a 200×magnification using a Leica DM6000B microscopy (Wetzlar, Germany). For immunofluorescence analysis of PPARγ2, threshold intensities for each analyzed field were determined. Then the number of pixels within the analyzed field above the threshold intensity was calculated and considered positively stained. The positively stained pixels as a percentage of the total pixels in five random fields per sample were calculated for the final analysis (25).

### Real-Time Quantitative PCR Analysis

According to the manufacturer’s instructions, the total RNA from the right tibiae was extracted with the TRIzol RNA isolation reagent (Invitrogen, Carlsbad, CA). According to the instruction manual, the total RNA (2 µg) was reverse-transcribed to cDNA using the RevertAid First Strand cDNA Synthesis Kit (Thermo). The specific transcripts were quantified by real-time quantitative PCR using a FastStart Universal SYBR Green Master (Roche) and analyzed with an ABI-7500 Step One Plus RT-PCR system (Applied Biosystems) with the following thermal cycling parameters: 95°C for 10 min to activate DNA polymerase, 40 cycles of amplification (95°C for 15 s and 60°C for 60 s). Quantitative analysis was performed according to the ABI protocol. The threshold cycle (Ct) value was calculated from amplification plots. Relative quantification of gene expression was determined using the delta CT (ΔΔCT) method, with each sample being normalized to the expression of the housekeeping gene actin. Each sample was run in triplicate, and the expression level of each gene was expressed relative to the expression level of the gene in BMSCs of the TFE-treated OVX rats. The primers were designed and synthesized by Invitrogen based on the sequence published by GenBank. The primer sequences used in this study are shown in **Table 1**.

**Table 1:** Primer sequences for the quantitative reverse-transcription polymerase chain reaction.

Target genes	Forward (5’-3’)	Reverse (5’-3’)	Accession Number
Ucp1	CGGGCTTAAAGAGCGAGAGG	CTTGGATCTGAAGGCGGACT	NM_021833.5
Prdm16	CCAAGGCAAGGGCGAAGAA	AGTCTGGTGGGATTGGAATGT	NM_022114.4
FABP4	AAGGTGAAGAGCATCATAACCCT	TCACGCCTTTCATAACACATTCC	NM_001442.3
PPARγ2	ACCATGG TTGACACAGAGATGCCA	AGGAATGCGAGTGGTCTTCCATCA	NM_001145366.1
β-actin	CGAGTACAACCTTCTTGCAGC	CCATATCGTCCCAGTTGGTG	NM_007393.5

### Statistical Analysis

All statistical analyses were performed with IBM SPSS Version 26.0 (Armonk, NY). Data were expressed as mean ± SD. Two-way repeated measurement ANOVA was performed to determine the time by group interaction effects and/or time effects for the body weight, BMD, and marrow fat fraction. One-way ANOVA with Bonferroni’s multiple comparison test was used to detect the differences in other studied parameters among the three groups. *P* < 0.05 was considered to be statistically significant.

## Results

### Changes in Body Mass and Uterine Parameters

**Figure 2:**
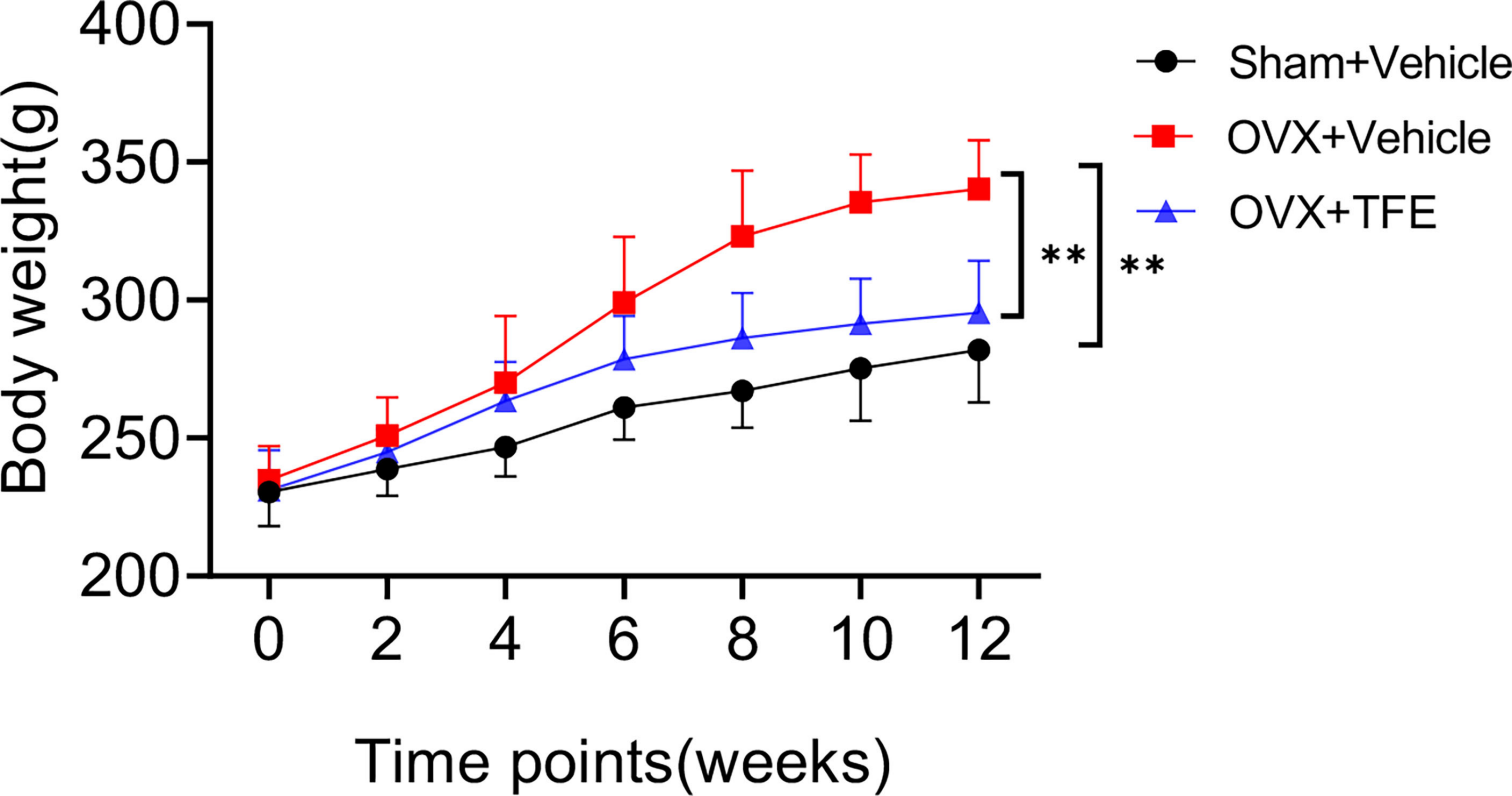
Body weights of rats in all treatment groups throughout the experimental period. Values are presented as mean ± SD (Sham, n = 11; OVX, n = 10; TFE, n = 11). OVX, ovariectomy; Sham, sham-operation; TFE, total flavonoids of Epimedium. *P* values represent Bonferroni-corrected *p*-values (two-way repeated-measures ANOVA). **P <0.001 between the OVX and Sham controls and between the OVX controls and TFE-treated group at week 12.

Of the initial 36 rats, 32 went through the whole study, whereas one rat in the Sham group, two in the OVX group, and one in the TFE group died of an adverse reaction to anesthetics. During the experiment, no adverse gastrointestinal effects (vomiting or diarrhea) or surgical complications (infection or wound dehiscence) were observed. **Figure 2** shows variations in the body weights of the various groups over time. At the beginning of the protocol, the body weights of the three groups of rats were not significantly different. All groups increased body weight across time points; a significant change among rats in the Sham, OVX, and TFE groups was first observed on week 4. On week 12, the body weight of OVX controls was 45.0% higher than at baseline, whereas the body weights of Sham and TFE rats were 22.6% and 28.2% higher, respectively. On week 12, the body weight of OVX controls was 20.6% and 15.2% higher than the body weights of Sham and TFE rats, respectively (all *P*<0.001).

**Figure 3:**
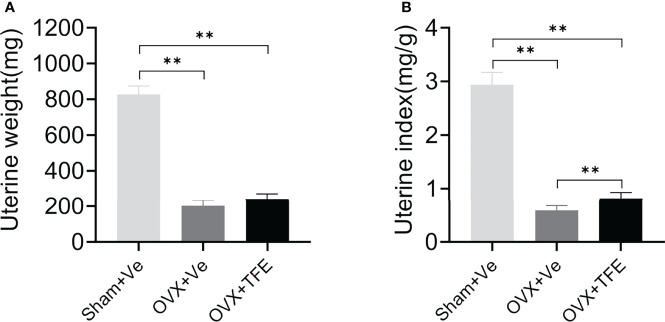
Effects of TFE treatment on **(A)** uterine wet weight and **(B)** uterine index. Data are presented as mean ± SD (Sham, n = 11; OVX, n = 10; TFE, n = 11). OVX, ovariectomy; Sham, sham-operation; TFE, total flavonoids of Epimedium; Ve, vehicle. **P<0.001 by One-way ANOVA with Bonferroni-correction.

The uterine weight and the uterine index were significantly reduced in the OVX group compared with the Sham group (all *P*<0.001), indicating that estrogen deficiency resulted in atrophy of the uterus of OVX rats. Treatment with TFE did not significantly increase the uterine weight of the OVX rats (*P* =0.064) (**Figure 3**).

### Changes in Bone Turnover Biomarkers and 17 β-Estradiol

**Figure 4:**
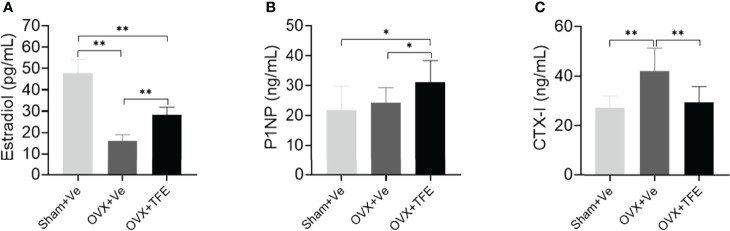
Effects of TFE treatment on **(A)** 17β-estradiol, **(B)** P1NP, and **(C)** CTX-I. Values are presented as mean (SD; Sham, n = 11; OVX, n = 10; TFE, n = 11). CTX-I, C-terminal telopeptides of type I collagen; P1NP, type 1 collagen amino propeptide; OVX, ovariectomy; Sham, sham-operation; TFE, total flavonoids of Epimedium; Ve, vehicle. TFE treatment inhibited bone turnover in OVX rats. Serum 17β-estradiol was significantly reduced while levels of CTX-I were higher in OVX rats than in Sham rats. *P<0.05 and **P<0.001 by One-way ANOVA with Bonferroni-correction.

The serum CTX-I level(*P*<0.001) was higher, and estradiol (*P*<0.001) was lower in the OVX rats than that in the Sham rats. There was no significant difference in the level of P1NP (*P*>0.05) between the OVX and Sham controls. The serum estradiol and the P1NP levels were increased when OVX rats were administrated with TFE treatment (all *P*<0.05). In contrast, TFE treatment decreased the serum CTX-I level of OVX rats (P<0.001) (**Figure 4**).

### TFE Increased BMD and Improved Trabecular Microarchitectures in OVX Rats

Lumbar spine BMD increased over time in the Sham group (+4.8% at week 6, *P >*0.05; +19.5% at week 12, *P <*0.001) and femur BMD (6 weeks +12.6%, *P <*0.001;12 weeks +24.4%, *P*<0.001). TFE treatment increased lumbar spine BMD (6 weeks, +3.6%, *P >*0.05) and (12 weeks, +12.2%, *P <*0.001) and femur BMD (6 weeks, +7.6%, *P >*0.05) and (12 weeks, +15.3%, *P <*0.001). In contrast, the BMD in the OVX control group slightly increased in the lumbar spine (+2.1% at week 6 and +5.9% at week 12 relative to baseline) and femur (6 weeks +6.0%; 12 weeks +7.8%). After OVX, the BMD was significantly decreased than the Sham group (all *P*<0.05). After TFE treatment, the BMD was increased as compared with the OVX but without statistical significance (all *P*>0.05) (**Table 2**).

**Table 2:** Changes in vertebral and femoral BMD in three groups of rats.

Parameters	Groups	BMD (g/cm^2^)		
		Week 0	Week 6	Week 12
Lumbar spine BMD	Sham+vehicle	0.184 (0.013)	0.193 (0.015)	0.220 (0.017)*^#^
	OVX+vehicle	0.183 (0.011)	0.187 (0.016)	0.194 (0.014)^a^
	OVX+TFE	0.184 (0.010)	0.191 (0.010)	0.207 (0.013)*^#^
Femur BMD	Sham+vehicle	0.243 (0.015)	0.273 (0.022)^a^	0.302 (0.016)*^#^
	OVX+vehicle	0.241 (0.010)	0.255 (0.015)	0.260 (0.028)^a^
	OVX+TFE	0.242 (0.014)	0.261 (0.022)	0.280 (0.021)*

Data are presented as mean (SD); Sham, n = 11; OVX, n = 10; TFE, n = 11.

TFE, total flavonoids of Epimedium; OVX, ovariectomy; Sham, sham-operation.

P values represent Bonferroni-corrected p-values.

a P <0.05 vs Sham+ vehicle (Bonferroni post-hoc test).

*P <0.05 vs week 0 and ^#^P <0.05 vs week 6 (paired multiple comparison test).

**Figure 5:**
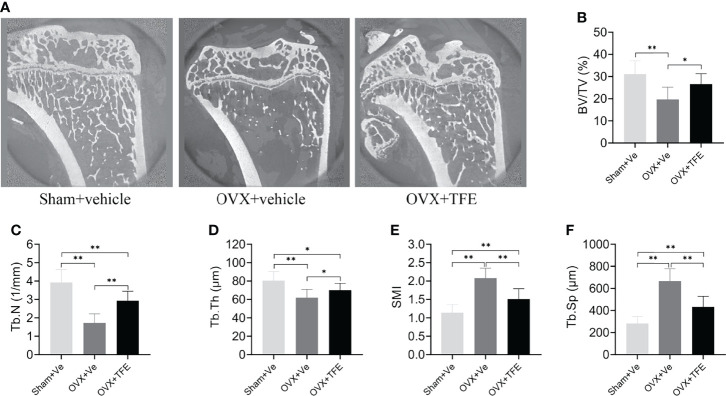
Effects of TFE treatment on bone trabeculae microstructure in the tibia in OVX rats. Sagittal views of micro-CT images at the proximal tibia region **(A)** showed deterioration of trabecular microstructure in OVX rats, and TFE treatment improved bone trabeculae microstructure. TFE-treated rats showed responses to the OVX-induced losses of **(B)** BV/TV, **(C)** Tb.N, **(D)** Tb.Th, **(E)** SMI, and Tb.Sp **(F)**. BV/TV, bone volume fraction; Tb. Sp, trabecular separation; Tb.N, trabecular number; Tb.Th, trabecular thickness; SMI, structure model index; OVX, ovariectomy; Sham, sham-operation; TFE, total flavonoids of Epimedium; Ve, vehicle. Data are shown as mean ± SD (Sham, n = 11; OVX, n = 10; TFE, n = 11). *P<0.05 and **P<0.001 between groups analyzed by One-way ANOVA with Bonferroni-correction.

Trabecular microarchitecture of the tibias was analyzed by micro-CT, and the results are shown in (**Figure 5**). Compared with the Sham group, BV/TV (-36.7%), Tb.N (-56.1%) and Tb.Th (-23.0%) were significantly decreased while Tb.Sp (+135.7%) and SMI (+82.5%, all *P <*0.001) were increased in the OVX group. Further compared with the OVX group, BV/TV (+35.0%), Tb.N (+70.3%) and Tb.Th (+13.1%) were significantly increased while Tb.Sp (-35.1%) and SMI (-27.4%, all *P*<0.05) were reduced in the TFE-treated rats, which indicated that TFE could restore the deterioration of trabecular microarchitectures.

### TFE Lowers Marrow Fat Expansion

After OVX, the marrow fat fraction was rapidly increased over time, by 34.7% at week 6 and 59.9% at week 12 relative to the baseline condition (all *P <*0.001) (**Table 3**). Compared with week 0, the marrow fat fraction was increased by 8.7% at week 6 (*P >*0.05) and 19.3% at week12 (*P <*0.001), respectively, in the OVX+TFE group. In addition, compared with the Sham group, it was found that at 6 weeks and 12 weeks, the marrow fat fraction of the OVX controls was increased by 26.9% (*P* =0.002) and 45.1% (*P <*0.001), respectively. The marrow fat fraction of the TFE-treated group was increased by 4.5% at week 6 (*P >*0.05) and 10.5% at week 12 (*P >*0.05) in comparison with the Sham controls, indicating that TFE administration significantly inhibited marrow adipogenesis caused by OVX.

**Table 3:** Changes in marrow fat fraction in three groups of rats.

Groups	Week 0	Week 6	Week 12
Sham + vehicle	14.5 (1.8)	15.6 (2.1)	16.2 (2.4)
OVX + vehicle	14.7 (2.5)	19.8 (2.9)^a^*	23.5 (3.4)^a^*^#^
OVX + TFE	15.0 (2.2)	16.3 (2.5)^b^	17.9 (2.7)^b^*^#^

Data are presented as mean (SD); Sham, n = 11; OVX, n = 10; TFE, n = 11.

TFE, total flavonoids of Epimedium; OVX, ovariectomy; Sham, sham-operation.

P values represent Bonferroni-corrected P values.

a P <0.05 vs Sham+ vehicle and

b P <0.05 vs OVX + vehicle (Bonferroni post-hoc test).

*P <0.05 vs week 0 and ^#^P <0.05 vs week 6 (paired multiple comparison test).

**Figure 6:**
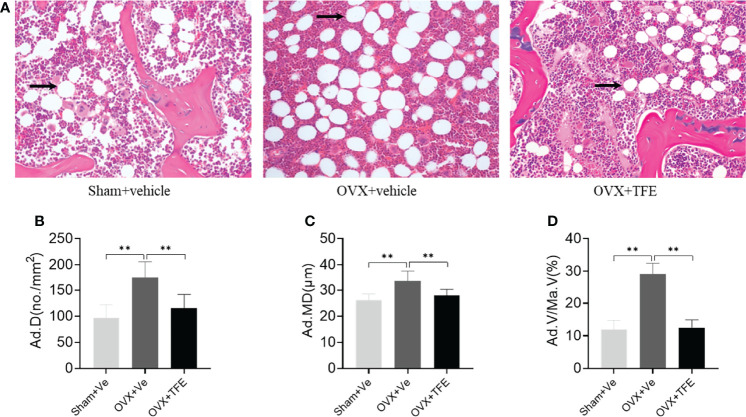
Changes in the marrow adipose **(A)** and adipocyte quantitative parameters **(B–D)**. Sections of decalcified bone were stained with H&E (×200). OVX markedly increased infiltration of marrow fat (arrow). This increase in marrow adiposity was restored by TFE treatment. Ad.D, adipocyte density; Ad.MD, adipocyte mean diameter; Ad.V/Ma.V, percentage adipocyte volume per marrow volume; OVX, ovariectomy; Sham, sham-operation; TFE, total flavonoids of Epimedium; Ve, vehicle. Data are shown as mean ± SD (Sham, n = 11; OVX, n = 10; TFE, n = 11). **P<0.001 between groups analyzed by One-way ANOVA with Bonferroni-correction.

Estrogen deficiency leads to marrow adipogenesis, shown as larger adipocyte size, increased adipocyte density, and increased percentage of adipocyte area. As expected, early TFE supplementation suppresses marrow fat accumulation as evidenced by decreased density (34.7%), mean diameter (16.7%), and area percentage (57.0%; all *P <*0.001) of adipocytes as compared with the OVX group (**Figure 6**).

### TFE Impacted Marrow Adipocyte Gene and Protein Expression

**Figure 7:**
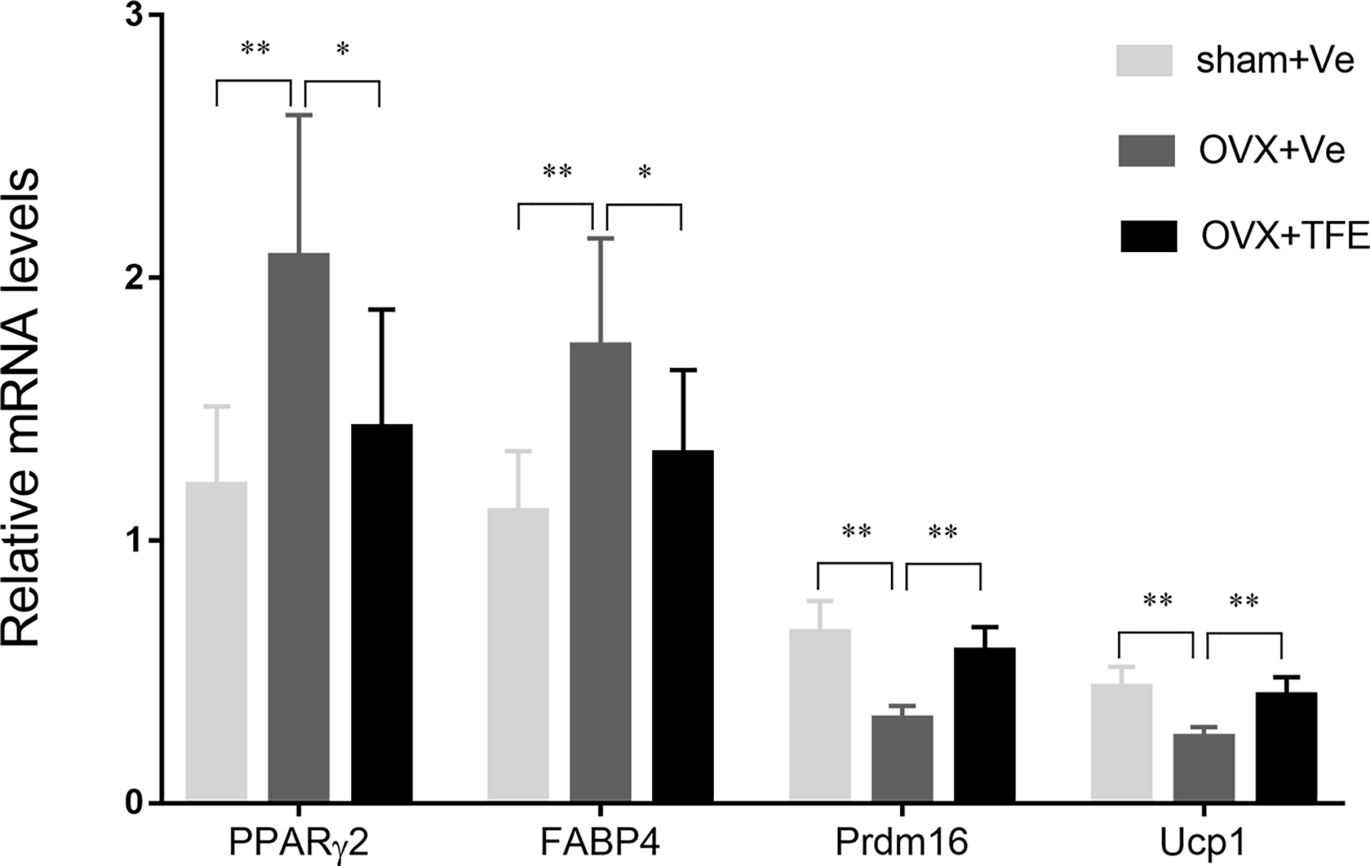
Effects of TFE treatment on marrow white and brown adipocyte gene markers in whole tibiae of OVX rats. Gene expression markers of Prdm16 and Ucp1 were normalized to FABP4 expression in the same sample. OVX, ovariectomy; Sham, sham-operation; TFE, total flavonoids of Epimedium; Ve, vehicle. Results are presented as means ± SD (Sham, n = 11; OVX, n = 10; TFE, n = 11). *P<0.05 and **P<0.001 between groups analyzed by One-way ANOVA with Bonferroni-correction.

OVX significantly increased white adipocyte transcript expressions of the PPARγ2 and FABP4 in intact tibiae. This effect was inhibited by TFE treatment. OVX significantly decreased brown adipocyte expressions of the transcription factor uncoupling protein 1 (Ucp1) and PR domain-containing 16 (Prdm16) (*P* < 0.05). TFE treatment for 12 weeks was associated with increased brown adipocyte markers expression in whole tibiae (**Figure 7**).

**Figure 8:**
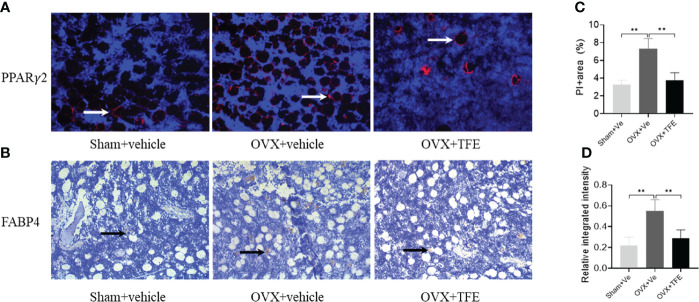
Representative microscopic features of PPARγ2 and FABP4 expression in bone marrow (200x magnification). Quantification of PPARγ2 expression by immunofluorescence **(A)** and FABP4 expression by immunohistochemistry **(B)** in the bone marrow showed a significant increase in PPARγ2 and FABP4 protein expression in proximal tibiae of OVX rats, which were restored to normal levels after TFE treatment. For PPARγ2, the positively staining pixels were presented as a percentage of the total pixels **(C)**; for FABP4, semi-quantification is expressed as the relative integrated intensity of intramarrow staining **(D)**. FABP4, fatty acid-binding protein 4; PPARγ2, peroxisome proliferator-activated receptor gamma-2; OVX, ovariectomy; Sham, sham-operation; TFE, total flavonoids of Epimedium; Ve, vehicle. Data are shown as mean ± SD (Sham, n = 11; OVX, n = 10; TFE, n = 11). **P<0.001 between groups analyzed by One-way ANOVA with Bonferroni-correction.

We next analyzed adipocyte marker expression at the protein level. As shown in **Figure 8**, OVX rats robustly increased PPARγ2 and FABP4 protein expression in proximal tibiae. Both Sham controls and TFE-treated groups had similar PPARγ2 and FABP4 positivity within the marrow space among marrow fat cells. These results suggest suppression of adipogenesis with TFE supplementation compared to the OVX vehicle. However, we could not detect Ucp1 or Prdm16 protein expression in all groups.

## Discussion

A series of results showed that TFE could induce bone marrow stromal cells (BMSCs) to differentiate into osteoblasts, enhance their activity, and inhibit bone catabolism and bone resorption produced by osteoclasts (26, 27). In this study, we first evaluated the effects of TFE on bone metabolism. Similar to previous data (17, 19, 28), TFE has an anti-osteoporotic effect as evidenced by bone turnover biomarkers, BMD, and bone trabecular microstructures.

Both osteoblasts and adipocytes are derived from BMSCs, and it is adequate to find agents that promote osteogenesis and inhibit adipogenesis differentiation of BMSCs. Our previous study verified that early TFE treatment could effectively restore marrow adiposity in estrogen-deficient rats (29). The current study further observed that TFE could markedly reduce marrow white fat markers. There are also a series of studies directly targeting bone marrow adipose tissue to treat osteoporosis, such as the antagonization of adipocyte-related transcription factors or targeting cytokines. Bisphenol A diglycidyl ether reduced marrow adiposity and increased bone mass in male C57BL/6 mice (30). Besides, inhibiting sclerostin could significantly reduce marrow fat expansion and improve bone mass in OVX models (31).

Treatment of marrow adipocytes with rosiglitazone, triiodothyronine, or thyroid hormone receptor beta-specific agonist markedly increased expression of several brown/beige adipose markers, indicating bone marrow fat has a mixed brown/white adipose tissue phenotype (9, 32). However, TFE regulation of marrow brown adipose has not previously been examined. In this work, we first analyzed the mRNA expression of adipocyte markers in the total tibia. Marrow brown adipocyte gene markers were upregulated in the total tibia in response to TFE treatment, accompanied by decreased marrow fat fraction, percentage of adipocyte area, and adipocyte density and diameter. Interestingly, the beneficial effect of bone marrow fat on the regulation of bone mass is presented through the expression of brown adipocyte gene markers (Ucp1, Prdm16), and this ability diminishes with a decrease in metabolic capacity due to age or diabetes (9). In consistent with our reports, *in vitro* studies have shown that rat bone marrow adipocytes express the adipocyte gene markers PPARγ and CCAAT/enhancer-binding protein-α, but not the brown fat gene markers Ucp1 and Cidea (33). Lineage tracing of Ucp1 expression in mTmG reporter mice was absent in bone marrow fat tissue, and stimulation with β3-agonists could not induce Ucp1-Cre expression in bone marrow fat (34).

At the level of RNA transcript, in solid support of marrow adipose brown potential, adipocytes in the proximal tibia have been reported to express brown fat markers, particularly Ucp1 (9–12). To further evaluate whether the increase in expression of brown transcripts such as Ucp1, Prdm16 in the TFE-treated group supported a corresponding protein expression change, Ucp1 and Prdm16 immunostaining on bone sections were conducted. In consistence with several other studies (3, 35), we were unable to detect expressions of protein related to brown fat markers within the tibia. In contrast, a previous *in vivo* study done by Nishio et al. (36) showed that a multilocular appearing cell in murine vertebral bone marrow expressed Ucp1 protein with immunostaining evidence.

The possible explanations for the significant discrepancies in existing literature might be attributed to the anatomical site of bone specimens, research designs, and methodological differences. First, the marrow adipose composition differs between anatomic sites. In both humans and rodents, marrow adipocytes are composed of two broad subtypes, namely regulated bone marrow adipose and constitutive bone marrow adipose, in which there are some critical differences (3, 37), including the composition of lipid content, response to pathophysiological stress, and levels of the adipogenic transcription factors. Cold exposure did not induce glucose utilization in the humeral or vertebral marrow adipose tissue compared to brown fat (38, 39), indicating that marrow adipose in humans is a metabolically active, insulin-sensitive fat tissue. Moreover, bone marrow adipose tissue did not express beta3-adrenergic receptor and Ucp1, both characteristic of brown fat (38); however, this result applied only to the mid-diaphysis of tibial and femoral marrow fat, which is mainly composed of constitutive marrow adipose tissue. Consistent with this, recent work by Craft et al. (35) using genetic ablation and lineage tracing of Ucp1 expressing cells has shown that marrow adipocytes do not express Ucp1 during development or after β3-agonist treatment in mice.

Another explanation could be that brown fat transcript was determined by RNA obtained from whole-mount skeletal preparations, which are highly inhomogeneous and especially susceptible to adipose tissue surrounding the skeleton. Furthermore, several studies indicated very low skeletal Ucp1 expression compared to brown adipose tissue (9, 12, 38).

The strength of our study is the characterizations of white and brown fat tissue with multiple methods, including *in vivo* VIBE MRI and gene expression analysis. The current study has some limitations that may need to be addressed by future research. First, our interpretation of this study may be limited by the gene expression analysis of RNA isolated from the whole tibia, mainly due to the difficulty in obtaining and maximizing the retention of bone marrow adipocytes during bone marrow isolation. Therefore, we separate RNA from the whole bone homogenate to represent all adipocytes. Furthermore, because the original cell population is a mixture of different types of cells, there may be deviations in the interpretation of the expression results of gene markers. Second, although our evidence-based *in vivo* VIBE MRI and gene expression analysis showed that TFE induces bone marrow white adipocytes to brown adipocytes, we did not confirm its effect on intact bone as the Sham and OVX rats were treated only with the vehicle and not TFE. Although OVX rats have a high rate of bone loss and prominent trabecular degeneration, their effect on the intact bone should be investigated in future studies. Third, our evidence suggests that the browning effect of TFE was associated with an increase in brown adipocytes. However, we are uncertain whether specific TFE-induced signaling pathways are involved in promoting brown adipocyte generation. In addition to its exclusive signaling pathway, the Wnt/β-catenin signaling pathway, TFE also directly regulates 11 estrogen-related targets and a set of target proteins to exert anti-osteoporosis effects (40). It is essential to determine what factors are involved in the browning effect of TFE on white adipose tissue through a signaling pathway array or gene chips. Finally, we used 3-month-old mice for OVX modeling in this study and started TFE intervention on the third postoperative day. Therefore, we are uncertain about the exact efficacy of TFE in aged mice and chronic osteoporosis.

In conclusion, our data demonstrated that TFE regulation of bone marrow adiposity in a rat model of estrogen deficiency-induced osteoporosis, at least in part, maintained the reciprocity of white and brown adipose tissue. TFE may induce browning of marrow white adipocytes due to increased expressions of brown transcripts Ucp1 and Prdm16 in the whole tibia; however, these results require further confirmation.

## Data Availability Statement

The datasets presented in this study can be found in online repositories. The names of the repository/repositories and accession number(s) can be found in the article/supplementary material.

## Ethics Statement

The animal study was reviewed and approved by Yueyang Hospital of Integrated Traditional Chinese and Western Medicine, Shanghai University of Traditional Chinese Medicine.

## Author Contributions

Study design: LC and G-WL. Study conduct: PL and DS. Data collection: RM and XS. Data analysis: LC and WY. Data interpretation: HN and S-XC. Drafting manuscript: LC and G-WL. All authors critically revised the manuscript and approved the final version of the manuscript to be submitted.

## Funding

This work was funded by the National Natural Science Foundation of China (No. 81874497).

## Conflict of Interest

The authors declare that the research was conducted in the absence of any commercial or financial relationships that could be construed as a potential conflict of interest.

## Publisher’s Note

All claims expressed in this article are solely those of the authors and do not necessarily represent those of their affiliated organizations, or those of the publisher, the editors and the reviewers. Any product that may be evaluated in this article, or claim that may be made by its manufacturer, is not guaranteed or endorsed by the publisher.
